# Decoding Adipose Tissue Phenotypic Switching: From Mechanisms to Computational Drug Discovery

**DOI:** 10.1007/s13679-026-00701-y

**Published:** 2026-03-23

**Authors:** Yuqing Ye, Ruxin Yin, Junjie Sun, Yuwei Dai, Di Zhao, Xiaoling Zou

**Affiliations:** 1 Hunan University of Chinese Medicine, Changsha, Hunan 410208 China; 2 Sir Run Run Shaw Hospital, Zhejiang University School of Medicine, Hangzhou, 310016 China; 3 The First Affiliated Hospital of Hunan University of Chinese Medicine, Changsha, Hunan 410007 China; 4 Xinhua Hospital, Shanghai Jiao Tong University School of Medicine, Shanghai, 200092 China

**Keywords:** Brown adipose tissue, 4-Hydroxybenzoic acid, Whitening, Plasticity, Obesity

## Abstract

**Purpose of Review:**

This review aims to explore the therapeutic potential of brown adipose tissue (BAT) to combat obesity and associated metabolic disorders by synthesizing the multifactorial influences and underlying mechanisms of BAT whitening and employing computational screening to identify promising candidate molecules for further investigation.

**Recent Findings:**

BAT whitening is characterized by the loss of thermogenic capacity, representing a critical aspect of adipose plasticity. Although diverse physiological and environmental triggers have been identified, the mechanistic interconnections underlying this process remain poorly understood. Emerging evidence supports an integrated view of these factors, and bioinformatic approaches now provide a valuable tool for the preliminary screening of potential intervention candidates.

**Summary:**

This review synthesizes current understanding on BAT whitening, from influencing factors to mechanistic pathways. Mitochondrial dysfunction appears to be a critical hub that could link diverse triggers to downstream metabolic and functional decline. Through bioinformatic screening, 4-hydroxybenzoic acid (4-HBA) is proposed as a candidate worthy of further study. Future work should prioritize experimental validation to clarify its mechanism and assess its translational potential.

## Introduction

Obesity, a prevalent chronic disease driven by metabolic dysfunction, represents a major global public health challenge [1, 2]. Adipose tissue, a central regulator of metabolic homeostasis, plays a key role in the process of obesity [3]. Notably, brown adipose tissue (BAT) has emerged as a promising therapeutic target for obesity and associated disorders not only for its canonical role in non-shivering thermogenesis but also for its regulatory effects on systemic glucose and lipid metabolism [4, 5]. Translating this therapeutic potential into clinical applications requires a comprehensive understanding of BAT physiology, appropriate research models, advanced detection technologies, and therapeutic strategies focused on either activating BAT or preventing its functional decline.

Specifically, regarding strategies to prevent functional decline of BAT, the central challenge is the process of “whitening”—a process in which thermogenic BAT loses its specialized phenotype and function due to cellular plasticity [6–8]. Accordingly, this review delineates the multiple influencing factors of BAT whitening and synthesizes their converging pathophysiological mechanisms. Mitochondrial dysfunction is widely observed and may be proposed as a potential initiating event in this process, which could trigger a cascade of downstream alterations. These include disrupted thermogenesis and lipid metabolism, activated stress and inflammatory responses [9–12]. These processes, along with dysregulated autophagy (including mitophagy) and degradation of the vascular and neural microenvironment, may contribute to a self-perpetuating cycle that drives the functional decline of BAT [13–16].

Moreover, to explore the therapeutic potential of targeting BAT whitening, we performed virtual screening and molecular docking, identifying the gut microbiota-derived metabolite 4-hydroxybenzoic acid (4-HBA) as a candidate for further study in BAT whitening. It has shown anti-inflammatory and antioxidant properties in experimental models, with limited evidence indicating a potential role in promoting adipose browning and improving metabolic homeostasis, possibly via the AMP-activated protein kinase (AMPK) -Dynamin-related protein 1 (DRP1) pathway [17, 18]. However, these findings are primarily based on computational and experimental models, which may not fully reflect human physiology. Their translational relevance is further limited by the absence of systematic dose-response and long-term safety studies in mammals [18]. While 4-HBA may be of interest for metabolic research, its therapeutic potential and precise mechanisms in obesity-related conditions require substantial further investigation.

## Translational Landscape of Brown Adipose Tissue

### Metabolic Regulation

Evolutionarily, BAT helped ancestors survive cold and hunger and now acts as a buffer for blood glucose and lipids [19, 20]. BAT contributes to overall health by boosting energy expenditure, enhancing glucose clearance, and exerting anti-inflammatory effects [3, 21]. Furthermore, it exhibits potential anticancer properties by competing with tumor cells for glucose, and influences female reproductive functions through the secretion of factors such as irisin, fibroblast growth factor 21 (FGF21), and adiponectin [22, 23]. Retrospective analyses link BAT presence to favorable metabolic profiles, such as improved glucose and lipid homeostasis, as well as lower hepatic fat content [24]. This is further associated with reduced cardiometabolic risk, particularly in obesity [25]. These findings underscore the potential relevance of BAT in metabolic health.

### Translational Model

The evolutionary relationship between human BAT and rodent models has been debated since the discovery of metabolically active BAT in adults. Early studies using cold-exposed young mice indicated a transcriptional similarity between human BAT and murine beige adipocytes [26, 27]. However, recent research demonstrates that the classical interscapular BAT in middle-aged mice closely models the key features of human supraclavicular BAT, supporting its translational relevance [28, 29].

Anatomical mapping studies have uncovered regional heterogeneity in human BAT [30]. Deep cervical BAT exhibits gene expression signatures closely resemble that of murine interscapular classical BAT, whereas BAT in intermediate regions displays moderate UCP1 expression, resembling murine inguinal beige fat [30, 31]. This distinction is further supported by infant adipose studies, where interscapular and perirenal BAT align with murine classical and beige signatures, respectively [32]. These findings underscore the necessity of using physiologically relevant models and considering depot-specific differences for translational BAT research.

### Technological Advances

In humans, BAT activity is primarily assessed through 18F-fluorodeoxyglucose positron emission tomography-computed tomography (^18^F-FDG PET/CT). Evidence from this modality demonstrates that women generally possess greater BAT volume or activity than men [33, 34], and that cold exposure robustly activates BAT to increase energy expenditure [35]. Recently, Creatine Chemical Exchange Saturation Transfer Magnetic Resonance Imaging (Cr-CEST MRI) has emerged as an alternative approach. By exploiting the chemical exchange properties of creatine, Cr-CEST MRI sensitively detects cold-induced BAT activation with high concordance to ^18^F-FDG PET/CT. Given its advantages of no ionizing radiation, high reproducibility and multimodal capability, Cr-CEST MRI represents a promising tool for investigating BAT pathophysiology in metabolic disorders [36].

### Therapeutic Strategies

Current therapeutic approaches for targeting BAT primarily focus on activating its thermogenic function or preventing its whitening. A central pharmacological debate concerns whether β2- or β3-adrenergic receptors (β2-ARs vs. β3-ARs) predominate in human BAT activation. Evidence favoring β2-ARs includes its higher expression in human BAT and the ability of agonists salbutamol to promote glucose uptake and thermogenesis; however, its activation is associated with additional cardiovascular effects [37]. In contrast, the β3-AR agonist mirabegron activates BAT via the β3-AR/cAMP/UCP1 signaling cascade and improves glucose and lipid metabolism, yet its clinically effective doses non-selectively activate other β-ARs, thereby increasing cardiovascular risk, while lower doses show limited efficacy [38, 39]. Therefore, clarifying receptor-specific roles and developing tissue-selective delivery systems are crucial for safer BAT-targeted therapies.

Another strategy aims to prevent the whitening of BAT. Supporting evidence from various clinical and experimental studies, summarized in Table 1, highlights the potential of the adrenergic agents mentioned above and specific anti-whitening compounds. Promising agents include the dual GIP/GLP-1 receptor agonist tirzepatide, which restores BAT morphology via improving mitochondrial dynamics, mitophagy, and biogenesis while suppressing endoplasmic reticulum (ER) stress and inflammation [40]; the senolytic combination of dasatinib and quercetin, which clears senescent cells, downregulates lipogenic genes, and enhances thermogenic gene expression to inhibit whitening [41]; the antidiabetic agent imeglimin counters whitening by inhibiting *de novo* lipogenesis and shifting mitochondrial substrate utilization toward lipids, independently of the gut microbiota [42]; the urate-lowering drug dotinurad reduces uric acid uptake and reactive oxygen species (ROS) production, thereby activating UCP1 and other thermogenic genes to restore BAT morphology [43], and the probiotic Roseburia hominis exerts protective effects partly through bacterial production of nicotinamide riboside, which elevates NAD^+^ to activate the Sirtuin1/mTOR pathway and subsequently leads to a reduction in lipid droplets along with the upregulation of thermogenic genes [44]. Together, these strategies highlight the therapeutic potential of preserving BAT identity and function for metabolic benefit.

**Table 1:** Summary of therapeutic studies targeting BAT thermogenesis and whitening prevention

Ref	Agent	Study Type	Study design	Population/Model	Sample size	Intervention/Dose	Comparator	Duration	Main outcomes
[37]	Salbutamol	Human clinical data	Single-center, randomized, double-blinded, crossover design	Healthy white Caucasian men; Age: 19–35 years; BMI: 19.2–26.5 kg/m²	Total: 10; EE analysis: 9	Salbutamol 250 µg IV + placebo PO	Salbutamol 250 µg IV + propranolol 80 mg PO; crossover randomization	Single dose; ~4 h per study visit	Salbutamol stimulates BAT glucose uptake, which correlates with increased energy expenditure
[39]	Mirabegron	Human clinical data	Randomized, double-blinded, placebo-controlled crossover study	Healthy lean men; Age: 18–30 years; BMI: 18–25 kg/m²	Total: 20; MRI analysis: 19	Single oral dose of mirabegron 200 mg	Placebo (oral);2-week washout༛crossover randomization	Single dose; ~3.5 h per study visit	Mirabegron reduces BAT fat fraction and increases skin temperature in the supraclavicular region
[40]	Tirzepatide	animal model	Controlled experimental design (induction + treatment phases); 8 groups (4 baseline + 4 treatment)	3-month-old female C57BL/6 mice; Induced phenotypes: obesity + type 2 diabetes + estrogen deficiency	Total: 80; 10 mice per group	Tirzepatide 10 nmol/kg/day, subcutaneous injection	Vehicle (Tris-HCl buffer, pH 8.0, 40 mM), subcutaneous injection	16 weeks (12-week induction + 4-week daily intervention)	Tirzepatide reverses BAT whitening; restores multilocular morphology; upregulates thermogenic markers/GLP-1R; improves mitochondrial function; reduces inflammation/ER stress
[41]	Senolytic cocktail (Dasatinib + Quercetin)	animal model	Controlled experimental design (induction + treatment phases); 4 parallel groups (2 baseline + 2 treatment)	8-week-old female C57BL/6NTac mice; Induced phenotype: obesity	Total: ~24–28;6–7 per group	Dasatinib 5 mg/kg + Quercetin 50 mg/kg, oral gavage	Vehicle (10% PEG400), oral gavage	24 weeks (24-week diet induction; intervention in weeks 16–24)	Senolytic cocktail prevents BAT whitening; increases thermogenic markers; reduces lipogenic genes; eliminates senescent cells
[42]	Imeglimin	animal model	Controlled experimental design (6-week treatment + microbiota ablation subgroup); 4 core groups (NCD, HFD+Vehicle, HFD+Imeglimin, HFD+Imeglimin+Antibiotics)	18-week-old male C57BL/6 mice; Induced phenotype: obesity	Total: ~38 (max); 8–10 (max) per group;	Imeglimin 300 mg/kg/day, oral administration	Vehicle (HFD feeding without imeglimin); NCD group; HFD+Imeglimin+Antibiotics (subgroup comparator)	6-week imeglimin treatment; Antibiotic co-treatment concurrent with imeglimin for 6 weeks	Imeglimin attenuates BAT whitening; decreases *de novo* fatty acid synthesis genes; inhibits mitochondrial basal respiration (pyruvate-stimulated) to enhance energy expenditure
[43]	Dotinurad	animal model	Controlled experimental design (induction + treatment phases); 4 parallel groups (2 baseline + 2 treatment)	8-week-old male C57BL/6 mice; Induced phenotypes: obesity	Total: ~62 (max); 15–16 (max) per group;	Dotinurad 50 mg/kg/day, oral administration	Vehicle (feeding without dotinurad)	20–22 weeks total (16–18-week diet induction + 4-week dotinurad treatment)	Dotinurad reverses BAT whitening; activates UCP1 (mRNA/protein upregulation) and thermogenic genes (Pgc-1α, Dio2); reduces BAT ROS production
[44]	Roseburia hominis	animal model	Controlled experimental design; 3 parallel groups (ND-PBS, HFD-PBS, HFD-R. hominis)	5-week-old male C57BL/6 mice; Induced phenotypes: obesity	Total: 15; 5 per group	R. hominis 10⁹ CFU/mouse, oral gavage, daily	Vehicle (PBS), oral gavage	15 weeks total (4-week co-housing + 11-week intervention)	Roseburia hominis inhibits BAT whitening; upregulates BAT thermogenic genes (Cidea, Ucp1, Pgc-1α, Dio2)

Abbreviations: *BAT* brown adipose tissue, *BMI* body mass index, *CFU* colony-forming units, *Cidea* cell death-inducing DFFA-like effector a, *Dio2* type II iodothyronine deiodinase, *EE* energy expenditure, *ER* endoplasmic reticulum, *GLP-1R* glucagon-like peptide-1 receptor, *HFD* high-fat diet, *IV* intravenous, *MRI* magnetic resonance imaging, *NCD* normal chow diet, *PBS* phosphate-buffered saline, *PEG400* polyethylene glycol 400, *Pgc-1α* peroxisome proliferator-activated receptor gamma coactivator 1-alpha, *PO* per oral, *ROS* reactive oxygen species, *Tris-HCl* tris(hydroxymethyl)aminomethane hydrochloride, *UCP1* uncoupling protein 1.

**Fig. 1:**
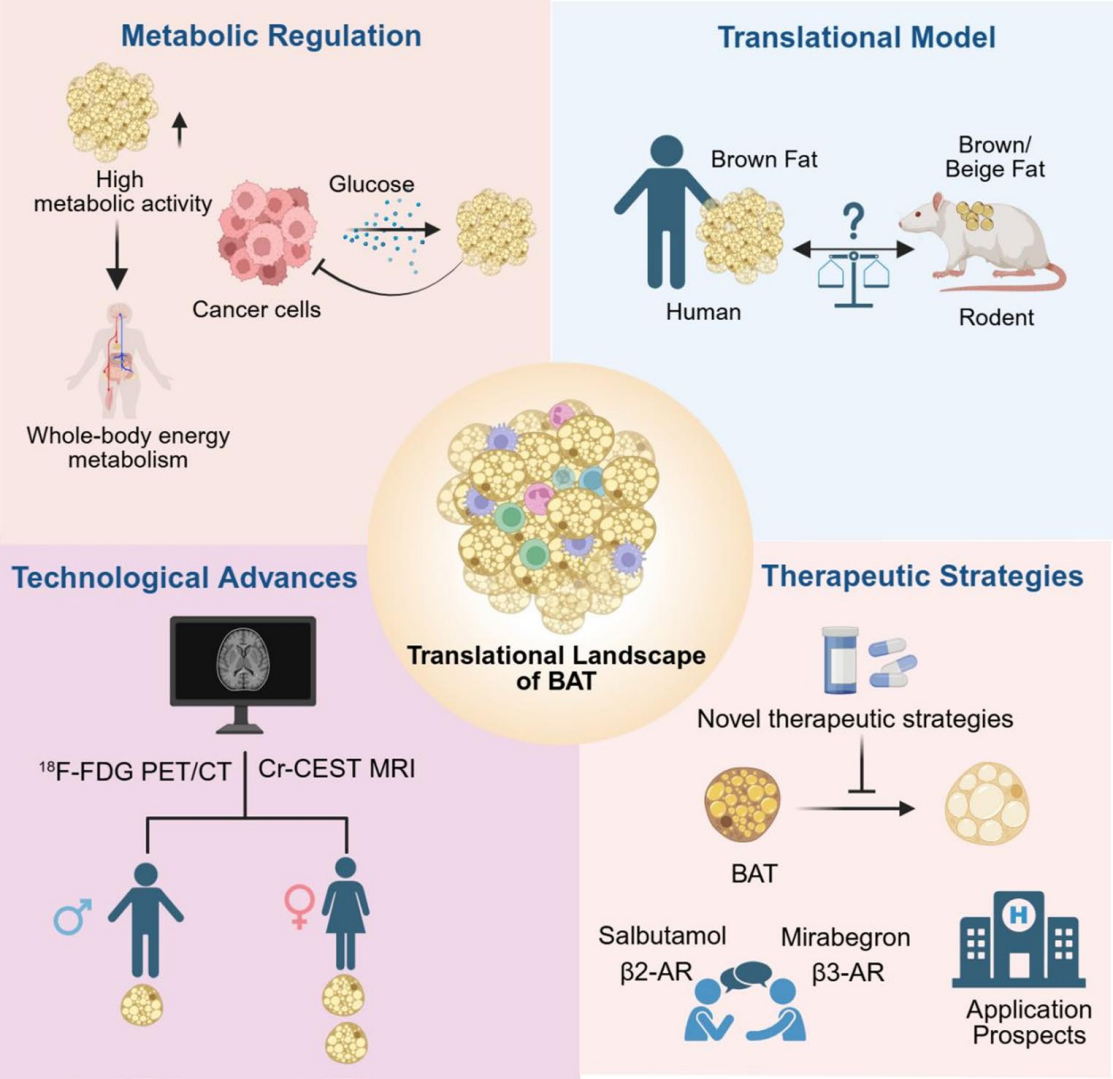
Translational landscape of brown adipose tissue (BAT). Schematic illustration of the translational landscape of BAT, with an emphasis on four domains: metabolic regulation (its multiple functions) [19–25]; Translational model (similarities between mouse and human BAT) [26–32]; technological advances (^18^F-FDG PET/CT and Cr-CEST MRI) [33–36]; and therapeutic strategies (activate existing brown fat or prevent its conversion into white fat) [37–44]

These key aspects of BAT—metabolic regulation, translational model, technological advances, and therapeutic strategies—are summarized in Fig. 1. Concurrently, a critical translational perspective necessitates acknowledging the predominant reliance on murine models, which limits direct extrapolation due to interspecies differences like circadian biology—thus highlighting the need for validation in diurnal models and human-centric studies to bridge the preclinical-clinical gap.

## Influencing Factors of Brown Adipose Tissue Whitening

**Fig. 2:**
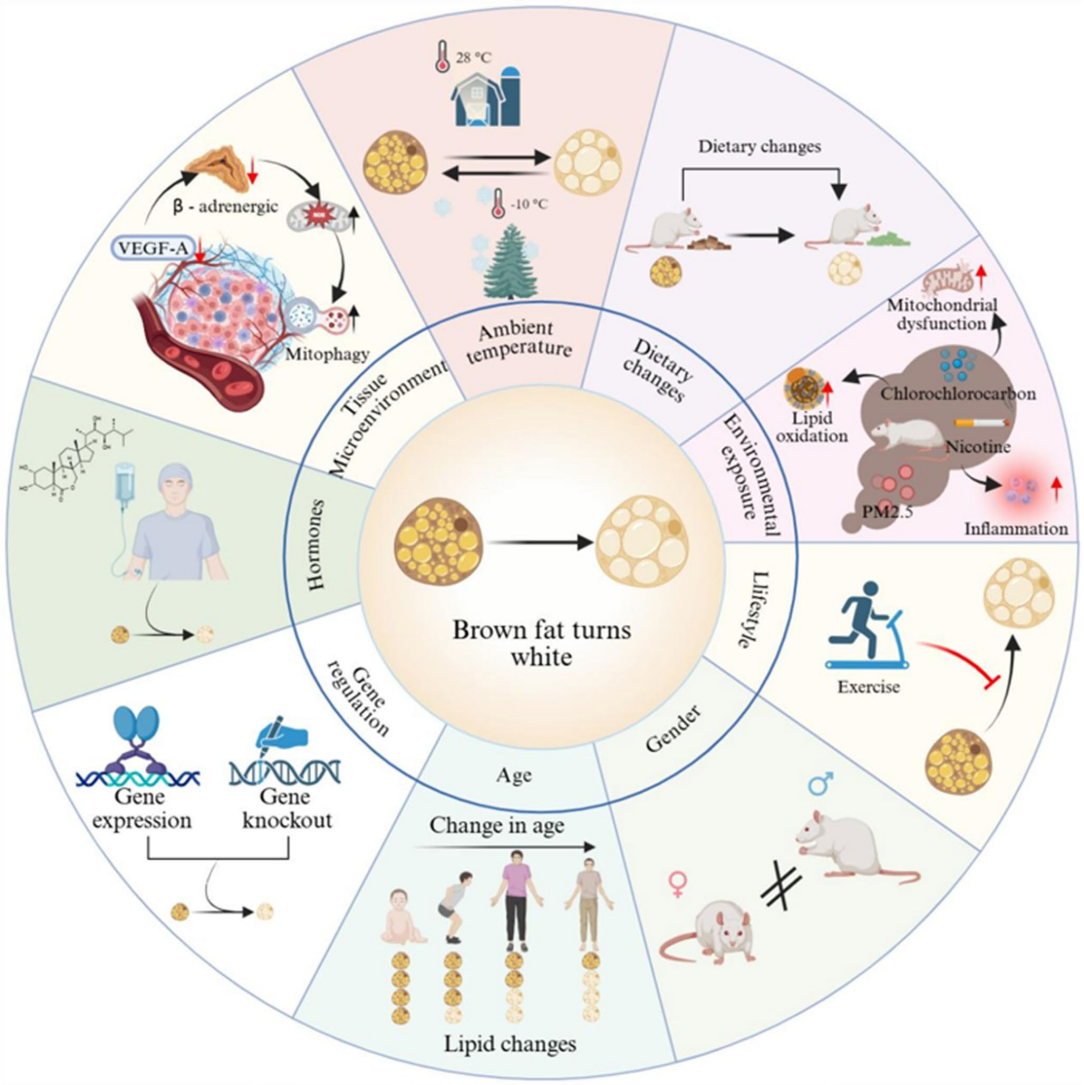
Overview of the various factors leading to the whitening of BAT. The schematic illustrates nine major factors influencing BAT whitening: warm temperature induces BAT whitening while cold exposure maintains its thermogenic capacity [9, 14, 45, 46, 48]; high-fat diets accelerate the process [47–49]; environmental exposures (e.g., DP, PM2.5, nicotine) induce whitening via inflammation, mitochondrial dysfunction, and altered lipid oxidation [50–52]; exercise suppresses whitening progression [53–55]; aging exacerbates whitening [25, 56]; gender differences impact metabolic responses [57, 58]; hormone signaling modulates the process [59–64]; tissue microenvironment changes (e.g., reduced VEGF-A) drive whitening through increased ROS and mitophagy [65, 66]; and gene regulation by multiple key genes critically influences the process [11, 67–70, 91–97]

As mentioned above, therapeutic strategies are directed toward either activating existing BAT or preventing its whitening. Understanding the process of BAT whitening is key to developing effective treatments. The following section systematically examines the key influencing factors of this process (Fig. 2), categorized as: (1) external inputs (ambient temperature, diet, environmental exposure, lifestyle); (2) intrinsic physiological modulators (age, gender, hormones); and (3) local executors (tissue microenvironment, genetic regulation). This progression from systemic triggers to local effectors sets the stage for identifying the convergent downstream pathways that drive the phenotypic switch.

### Ambient Temperature

Ambient temperature critically regulates metabolism by modulating the morphology and function of BAT. Reduced cold exposure dually suppresses energy expenditure, diminishing both thermogenic demand and intrinsic capacity. Since the 1960 s, increased indoor temperatures due to widespread climate control have lowered cold exposure, contributing to a decline in human thermogenic capacity and BAT activity [71]. Additionally, energy expenditure and cold-induced thermogenesis are higher in winter than in summer, with BAT metabolic activity peaking during the colder months [72].

In rodents, exposure to warm conditions (27–30 °C) promotes BAT whitening, characterized by reduced expression of thermogenic genes and proteins, increased macrophage infiltration and crown-like structure formation [9, 45]. This shift is associated with suppressed sympathetic activity, decreased norepinephrine turnover, downregulated tyrosine hydroxylase expression, and enhanced mitochondrial degradation alongside reduced mitochondrial mass [14, 67]. Conversely, cold exposure counteracts whitening, reducing triglyceride content and downregulating lipogenic factors in BAT [46].

### Diet

Altered dietary composition is a key driver of BAT whitening in rodent [10]. Chronic high-fat diet (HFD) feeding promotes lipid deposition and whitening in BAT, characterized by the upregulation of adipogenic and pro-inflammatory genes, increased ER stress, and downregulation of thermogenic genes [47, 48]. Short-term HFD rapidly induces lipid accumulation via fatty acid influx-driven endocannabinoid synthesis, which blunts norepinephrine signaling and promotes mitochondrial fusion, thereby impairing fatty acid oxidation [49]. Similarly, a high-fructose diet (HFrD) induces this phenotype, which can be counteracted by PPAR-α agonists through enhanced thermogenesis, β-oxidation and angiogenesis [15]. In rats, both HFD and high-fat/high-sucrose diets (HFSD) impair UCP1-mediated oxidation and shift metabolism toward fatty acid esterification and triglyceride synthesis in BAT, thereby exacerbating obesity [73, 74].

Dietary interventions further influence BAT plasticity: positive modulators, including coffee, tart cherry supplementation, and omega-3 fatty acids, promote mitochondrial biogenesis, upregulate thermogenic gene expression, and reduce lipid infiltration [75–77]. Conversely, negative modulators such as palm and interesterified palm oil suppress thermogenic markers and promote inflammation, thereby accelerating BAT whitening [78].

### Environmental Exposure

Environmental exposure significantly impacts BAT, inducing metabolic changes with obesity and related diseases. Chronic exposure to fine particulate matter (PM2.5) induces BAT whitening in rodents via mitochondrial dysfunction, ER stress and inflammation [50]. Similarly, chemical pollutants such as the flame retardant dechlorane plus (DP) and the fungicide prothioconazole (PTC) promote lipid accumulation and whitening in mice [51, 79]. Endocrine disruptors like bisphenol S (BPS) can trigger whitening through glucocorticoid receptor-mediated epigenetic alterations [68], while prenatal exposure to nicotine or polystyrene nanoplastics (PSNPs) results in impaired BAT function in male offspring [52, 80]. Notably, combined ozone and heat exposure exerts a synergistic effect on whitening, linked to activation of the stress axis and increased stress hormone secretion [81].

### Lifestyle

Early-life exercise contributes to long-term metabolic health by suppressing BAT whitening, which helps prevent obesity and diabetes through preserved muscle mass and improved lipid metabolism [53]. Since BAT whitening and mitochondrial protein downregulation rapidly progress when exercise ceases, consistent physical activity is essential to sustain these protective effects [54]. Exercise-derived or dietary lactate counteracts BAT whitening in obesity by activating the G protein-coupled receptor 81 (GPR81)-Ca2+/calmodulin-dependent protein kinase (CaMK) pathway, which synergizes with adrenergic signaling to upregulate UCP1 expression and mitochondrial DNA (mtDNA) content [55]. Additionally, the exercise-induced myokine irisin exerts protective effects on BAT, contributing to maintaining its thermogenic capacity and resisting whitening [82, 83]. Furthermore, exercise can also mitigate whitening through sympathetic activation, enhanced mitochondrial function, and promoted BAT endocrine activity through the release of 12,13-dihydroxy-9Z-octadecenoic acid (12,13-diHOME) [84].

### Age

Aging serves as a key driver of BAT functional decline and whitening. Large-scale human studies reveal a progressive, age-dependent decline in both BAT metabolic activity (measured via ¹⁸F-FDG PET/CT) and overall abundance [25, 56]. In rabbits, age-related BAT whitening involves progressive loss of thermogenic and mitochondrial function, enhanced autophagy, vascularization, innervation and immune cell infiltration [16]. Long non-coding RNAs (LncRNAs) participate in this process, with differential LncRNA mainly participating in purine metabolism, Wingless and Int-1(Wnt), Peroxisome Proliferator-Activated Receptor (PPAR), Cyclic Guanosine Monophosphate/cGMP-Dependent Protein Kinase (cGMP/PKG), and lipid metabolism pathways [85]. Besides, circular RNA (circRNA)-and lncRNA-mediated competing endogenous RNA (ceRNA) networks regulate age-related whitening through the Mitogen-Activated Protein Kinase (MAPK) and Rat sarcoma virus oncogene homolog (Ras) signaling pathways [86]. Temporal transcriptomic profiling further delineates two distinct phases of BAT whitening: an early stage marked by alterations in angiogenesis, mitochondrial function, and thermogenesis, followed by a late stage characterized by diminished ATP synthesis, reduced fatty acid oxidation, and a decline in progenitor differentiation potential [87].

In lambs, postnatal BAT whitening is associated with the downregulation of Vascular Endothelial Growth Factor A (VEGFA) and thermogenic genes, as well as the upregulation of Cytochrome P450 Family 1 Subfamily A Member 1 (CYP1A1) [88]. In mice, aging promotes BAT whitening through T cell-derived Interferon-gama (IFN-γ) [89]. Furthermore, α-lipoic acid ameliorates age-related metabolic decline and enhances mitochondrial fatty acylation to restore thermogenesis and glucose homeostasis [90].

### Gender

Gender significantly impacts brown fat metabolism, showing different adaptations in various physiological states. In humans, BAT is markedly more prevalent in women than in men, and its age-related decline in both metabolic activity and abundance is more pronounced in men, whereas women exhibit a relatively attenuated reduction over time [25, 91]. Similarly, male mice exhibit morphological abnormalities and thermogenic dysfunction in brown fat during aging, while female rodents better maintain thermogenic capacity [57]. Maternal high-fat diet affects offspring with sex differences, causing brown fat whitening, inflammation, and abnormal oxidative phosphorylation in male offspring, while females show different metabolic adaptations [58].

### Hormones

Emerging evidence indicates that glucocorticoid signaling (GCS) induces BAT whitening through multiple pathways, including miR-21-5p-mediated suppression of thermogenic genes, leptin resistance induction, BTG1-dependent autophagy, and disruption of mitochondrial homeostasis [59–61]. Estrogen inhibits whitening by modulating hypothalamic ceramide and ER stress to preserve BAT function [62], while androgen promotes lipid accumulation and the downregulation of UCP1 [63]. Hyperprolactinemia reduces thermogenic genes expression and may cause mitochondrial dysfunction and inflammation in BAT [64].

### Tissue Microenvironment

BAT exhibits high vascularity and is regulated by the sympathetic nervous system. VEGF-A absence reduces capillaries, lowers β-adrenergic signaling, increases mitochondrial reactive oxygen species (mtROS) and promotes mitophagy [65]. Intravitreal injection of anti-VEGF antibodies in mice reduces brown fat VEGF levels, increases lipid accumulation, lowers vascular density and downregulates mitochondrial genes, causing whitening [66].

### Genetic Regulation

The maintenance of brown fat function and identity is critically dependent on a multi-layered gene regulatory network. Alterations such as gene knockout or overexpression disrupt this network, leading to BAT whitening and functional decline. Negative regulators, including Carbohydrate-responsive element-binding protein β (ChREBP-β) and the histone demethylase KDM5A, suppress BAT identity when overexpressed by repressing mitochondrial dynamics and thermogenic gene programs, respectively [11, 68].

Conversely, the loss of positive regulators—including Transcription Factor EB (TFEB) [67], Mitochondrial Transcription Factor A (TFAM) [69], Estrogen-Related Receptor γ (ERRγ) [92], the RNA-binding protein Family with sequence similarity 195 member A (FAM195A) [93], Adipose Triglyceride Lipase (ATGL) [94], the miRNA-processing enzyme Dicer [95], UCP1 [96, 97], Apolipoprotein O (APOO) [98], and the secreted protein Noggin [70]—disrupts key cellular processes. Deficiency in any of these factors disrupts essential cellular processes—such as autophagic flux, lipid catabolism, mitochondrial function, oxidative stress response, and thermogenic gene expression—converging on the common pathological outcome of BAT whitening. Therefore, the stability of the brown adipocyte phenotype is not governed by a single gene but emerges from the integrity of this integrated, multi-tiered regulatory system, wherein an imbalance at any level can precipitate systemic functional collapse.

## Integrated Molecular Mechanism of BAT Whitening

Moving beyond cataloguing individual factors, we propose that BAT whitening is orchestrated through a self-reinforcing, mitochondria-centric vicious cycle. Within this framework, mitochondrial dysfunction acts not merely as one of many defects, but as a critical integrative hub and a principal cellular executor of the phenotypic switch. It serves as both the common downstream consequence where diverse upstream insults (e.g., metabolic stress) converge, and a key proximate cause that initiates and amplifies the pathological cascade.

Within this framework, the contributors to BAT whitening largely converge to directly initiate and exacerbate mitochondrial dysfunction. This dysfunction manifests as impaired electron transport chain activity, disrupted dynamics, and structural damage, driving increased mtROS production and cellular stress [99–101]. Crucially, this damage establishes a self-reinforcing loop, where mitochondrial-derived signals further exacerbate the initial dysfunction, locking the cell in a state of progressive energetic deficit [102–105].

Downstream, this pathophysiological cascade is linked to the suppression of the thermogenic gene program and a loss of brown adipocyte identity [106, 107], alongside disruptions in lipid metabolism, such as reduced fatty acid oxidation and lipolysis [104, 108, 109]. Furthermore, attenuated thermogenic signaling is associated with the activation of cellular autophagy, particularly mitophagy. This selective degradation of mitochondria can lead to a reduction in mitochondrial mass [13]. Thus, functional impairment and quantitative loss synergistically deepen the metabolic crisis.

These cellular disturbances are accompanied by secondary tissue-level alterations, including impaired angiogenesis (promoting a hypoxic microenvironment), local inflammatory activation, and oxidative stress-associated sympathetic denervation, which collectively disrupt thermogenic activation [109–111]. This integrated perspective highlights whitening as a progressive, self-reinforcing cycle of energetic deficit and functional decline.

In summary, this integrated framework recasts BAT whitening as a hierarchical, vicious cycle initiated by diverse factors, executed and amplified through mitochondrial dysfunction, and often accompanied by downstream cellular and tissue-level sequelae. Clarifying this mechanism underscores that preserving mitochondrial function is not merely supportive but central to intercepting the entire pathological cascade.

## Integrative Bioinformatic Exploration Highlights 4-Hydroxybenzoic Acid as a Candidate for Therapeutic Investigation

To identify potential therapeutic targets for mitigating the whitening of BAT, we focused on the key regulatory factors involved in this process as outlined previously. Eleven proteins critically associated with adipocyte plasticity and metabolic reprogramming were selected for analysis: MLXIPL (encoded by ChREBP-β), TFEB, TFAM, ERRγ, KDM5A, FAM195A, ATGL, DICER, UCP1, APOO and Noggin (see Methods for detailed selection criteria and rationale). Functional categorization revealed that these 11 proteins cluster into three distinct classes—transcriptional/epigenetic regulators (MLXIPL, TFEB, TFAM, ERRγ, KDM5A, FAM195A), metabolic enzymes (ATGL, DICER), and transporters and secreted proteins (UCP1, APOO, Noggin)—collectively representing the major regulatory layers of BAT whitening, ranging from nuclear transcriptional control and metabolic substrate switching to extracellular signal integration. To ensure clinical relevance, the three-dimensional crystal structures of these human target proteins were obtained from the UniProt database. Structural preparations were performed using AutoDockTools, including hydrogen addition, charge assignment, and atom type definition [112]. Molecular docking-based virtual screening was subsequently conducted against the U.S. Food and Drug Administration (FDA)-approved compound library using AutoDock Vina. Resulting docking poses were visualized and analyzed using PyMOL.

**Fig. 3:**
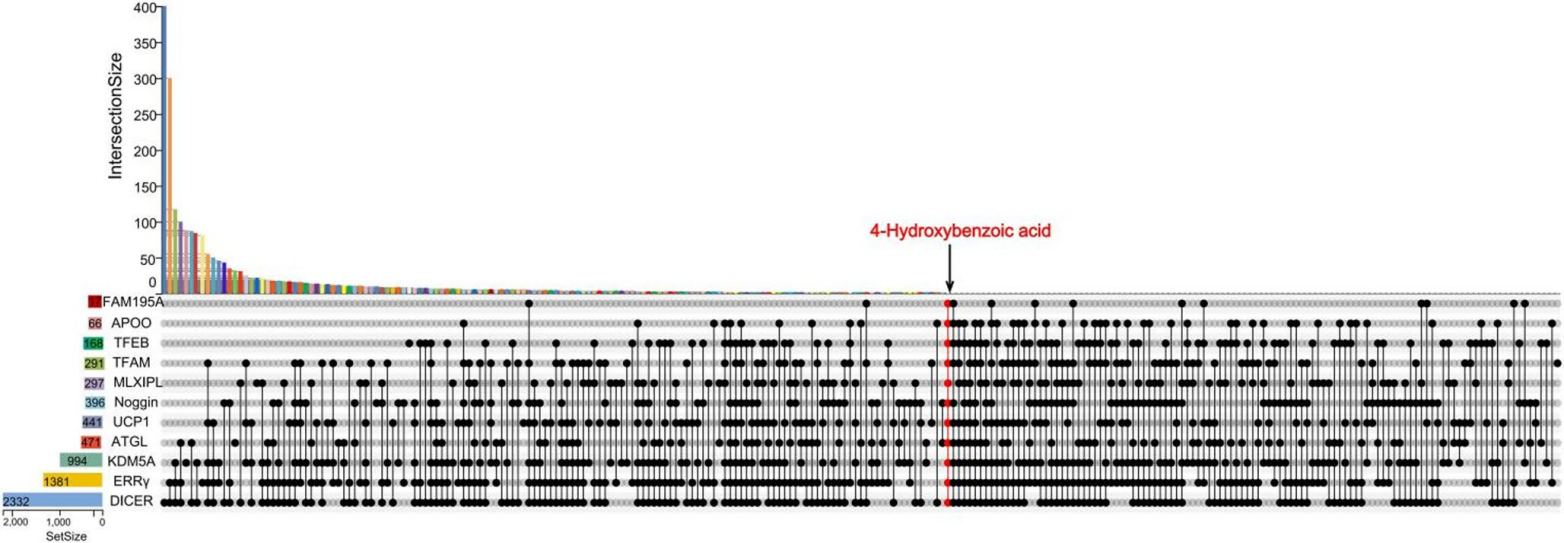
Identification of 4-Hydroxybenzoic acid by intersection analysis. An UpSet plot displays the intersections of hit compounds (predicted binding energy ≤ -5.0 kcal/mol) from an FDA-approved library across 11 target proteins. Each vertical bar represents the intersection for the combination of proteins connected by the dots below. The red column highlights the sole intersection of all 11 proteins, which exclusively contains 4-Hydroxybenzoic acid

**Fig. 4:**
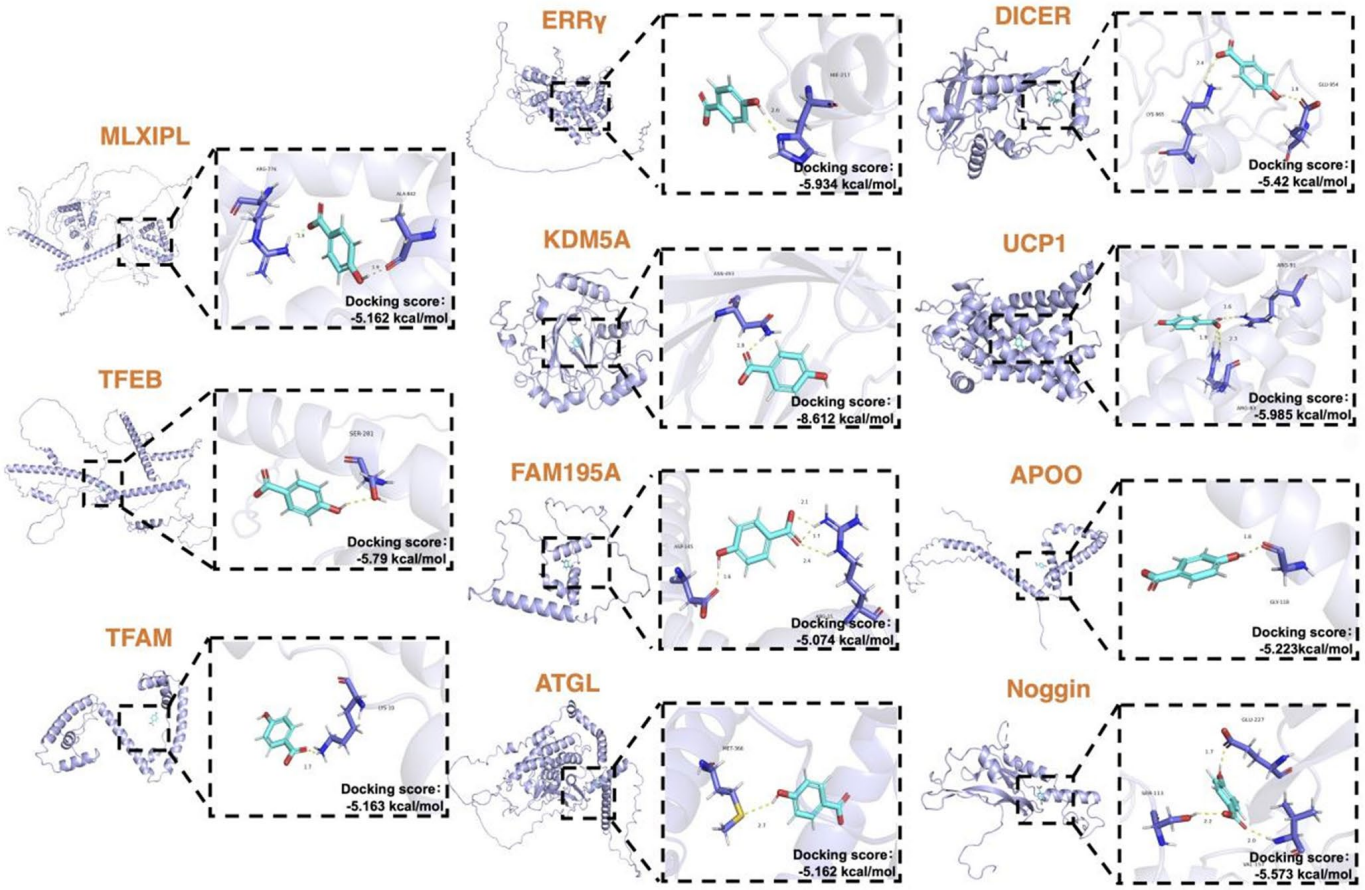
Predicted binding modes of 4-Hydroxybenzoic acid with 11 target proteins. Molecular docking results showing the binding poses of 4-Hydroxybenzoic acid (displayed as cyan sticks) within the binding pockets of 11 target proteins (MLXIPL, TFEB, TFAM, ERRγ, KDM5A, FAM195A, ATGL, DICER, UCP1, APOO and Noggin), each represented in blue-purple cartoon representation. Putative hydrogen bonds are depicted as yellow dashed lines. Key interacting residues are shown as sticks, with oxygen atoms colored red and nitrogen atoms colored dark blue. The structural visualization was generated using PyMOLchematic illustration of the Translational L

Using a binding energy threshold of ≤ − 5 kcal/mol—a criterion widely accepted in molecular docking studies as indicative of strong and stable binding [113–116], we initially identified candidate compounds exhibiting high binding affinity for each individual protein. Intersection analysis identified 4-Hydroxybenzoic acid (4-HBA) as the sole compound that exhibited significant binding affinity for all eleven target proteins (Fig. 3). Visualization of the molecular docking models (Fig. 4) indicated that 4-HBA forms stable molecular interactions with key residues within the binding pockets of each protein.

4-HBA, an aromatic organic acid derived from benzoic acid, is one of the most abundant phenolic compounds produced by the human gut microbiota. It is generated endogenously via tyrosine metabolism and exogenously through microbial fermentation of dietary polyphenols present in foods such as green tea, berries, olives, and coconut [117, 118]. Preliminary evidence suggests 4-HBA may exhibit multiple bioactive properties, including antioxidant, anti-inflammatory, and antimicrobial effects, and been implicated in the regulation of gut microbial composition by promoting beneficial bacteria and suppressing harmful species [17]. Furthermore, 4-HBA serves as a precursor for coenzyme Q10 and has been shown to exhibit neuroprotective and cardioprotective functions in previous studies [119, 120]. Its metabolic fate involves conversion into several biologically active derivatives, including salicylic acid, vanillic acid, gallic acid and ellagic acid. This biotransformation is mediated through pathways involving both gut microbiota and host enzymatic activities [18]. Emerging evidence suggests that certain metabolites of 4-HBA, including vanillic acid and ellagic acid, can promote adipose tissue thermogenesis and mitigate metabolic disturbances in models of obesity [121, 122]. Besides, a study has reported that 4-HBA promotes adipose tissue browning in diet-induced obese mice through AMPK-DRP1 activation [18].

However, several important limitations of the current research on 4-HBA must be acknowledged. The existing evidence remains limited to short-term rodent studies, which lack clinical correlation. Furthermore, the pharmacokinetics and tissue-specific bioavailability of 4-HBA in mammals are still unclear, while its potential off-target effects and long-term safety profiles remain largely unexplored. Finally, the ecological relevance and generalizability of its gut microbiota-modulating effects across diverse human populations require further validation.

While these preliminary findings cautiously propose 4-HBA as a compound of interest for metabolic research, substantial additional studies are required to comprehensively evaluate its therapeutic potential and mechanistic underpinnings in obesity-related metabolic conditions.

## Conclusions and Discussion

Given the crucial role of BAT in regulating systemic energy metabolism, preventing its whitening represents a promising therapeutic target against obesity and related metabolic diseases [6, 24]. This review systematically summarizes the translational landscape of BAT and provides a comprehensive overview of multiple factors influencing BAT whitening and underlying mechanisms (Table 2). Furthermore, we integrate these findings to propose that whitening progresses through a self-reinforcing cycle centered on mitochondrial dysfunction as an integrative hub, leading to progressive adipose tissue dysfunction.

**Table 2:** Summary of the factors and potential mechanism linked to BAT whitening

Ref	Mouse model/exp. condition	Phenotypes and mechanism
Ambient Temperature		
[9]	12-week-old female C57BL/6J mice were kept at 28 °C or at 6 °C for 10 days	Warm conditions: macrophage infiltration↑ crown-like structure formation↑
[14]	A/J mice were raised under either 22–30 °C	Warm conditions: thermogenic program↓ tyrosine hydroxylase expression↓ norepinephrine turnover↓ sympathetic activity↓
[45]	10-week-old male C57BL/6J mice were kept at 27 °C or at 22 °C	Warm conditions: Ucp1/Pgc-1α/Elovl3↓
[67]	The mice were housed under either 22–30 °C for 7 days	Warm conditions: mitochondrial biogenesis markers↓ mitochondrial degradation markers↑
Diet		
[15]	3-month-old male C57BL/6 mice were fed with CD、HFD or HFrD	HFrD-induced BAT whitening was reversed by PPAR-α agonists: thermogenesis↑β-oxidation↑ VEGFA-driven angiogenesis↑
[47]	3-month-old male C57BL/6J mice were fed with HFD for 12、16 or 20 weeks	HFD: Cidea/Plin1↑Pparα/Ucp1↓ pro-inflammatory (Tlr4/Nlrp3) ↑ER stress markers (Atf4/Chop/Gadd45)↑
[49]	15-week-old male C57BL/6J mice were fed with HFD for 1、3 or 7 days	HFD: substrate uptake↓ mitochondrial fusion↑ fatty acid oxidation↓
[73]	Male Wistar rats were fed with CD or HFSD for 8 weeks	HFSD: Ucp1-driven oxidation↓ lipolysis↓ tyrosine hydroxylase↓
[74]	8-week-old male Wistar rats were fed with HCD、HFD or HFSD for 12 weeks	HFD and HFSD diet: Ucp1-mediated oxidation↓ insulin receptor signaling↓ adipokine balance↓
Environmental Exposure		
[50]	Mice were exposed to chronic PM2.5 inhalation or PM2.5 intratracheal instillation	PM2.5: lipid oxidation↑ mitochondrial/ER stress↑ inflammation↑ insulin resistance↑
[68]	Mice were fed diets supplemented with low, medium, and high levels of BPS	BPS: KDM5A-dependent epigenetic reprogramming↑ estrogenic GR activation↑
[52]	Pregnant rats were randomly divided into a nicotine group and a control group	Prenatal nicotine exposure: brown fat-specific gene expression↓ mitochondrial structure/function↓
[80]	Pregnant C57BL/6J mice were randomly divided into a PSNPs group and a control group	Prenatal PSNPs exposure: lipogenic proteins (FASN, SREBP-1c/CD36/DGAT2)↑lipophagy pathway↓
[81]	8-week-old male C57BL/6 mice were divided into control, heat, ozone, and combined exposure groups	combined exposure: stress hormones glucocorticoid↑ epinephrine↑
Lifestyle		
[53]	4-week-old male OLETF (obese) rats underwent exercise followed by detraining; LETO (control) rats remained sedentary	Sustained exercise: skeletal muscle mass↑ post-training lipid metabolism↑
[54]	4-week-old male OLETF (obese) rats were randomized into non-exercise sedentary group or exercise groups, along with LETO (non-obese) rats	Exercise: fatty acid oxidation↑PGC-1α/UCP1↓
[55]	8-week-old male C57BL/6 mice were grouped by lactate supplementation and exercise	Exercise-derived or dietary lactate: GPR81-Ca2+/CaMK pathway↑UCP1↑ mtDNA↑
Age		
[16]	Male New England rabbits of different ages, including 1 day, 14 days, 1 month, 2 months, 3 months, and 4 months	Aging: Ucp1/mitochondrial gene↓ mtDNA↓ autophagy/vascularization/innervation/immune cell infiltration↑
[85]	Tianfu Black rabbits of different ages, including 0 day, 15 days, 85 days and 2 years	Aging: purine/Wnt/PPAR/cGMP/PKG/lipid metabolism pathways↑
[86]	Tianfu Black rabbits of different ages, including 0 day, 15 days, 85 days and 2 years	Aging: MAPK/Ras signaling pathways↑
[87]	New Zealand white rabbits of different ages, including 1 day, 3 weeks, 6 weeks and 12 weeks	Aging: Ucp1/Dio2/Pgc-1α↓leptin↑ progenitor cell differentiation↓
[88]	Newborn Romney lambs at 12–24 h and 2 days of age	Aging: VEGFA↓ thermogenic genes↓CYP1A1↑
[89]	3-month-old (young) and 18-month-old (old) male C57BL/6J mice	Aging: T cells-derived IFN-γ↑
[90]	10-week-old young and 76-week-old old mice	Aging: mitochondrial lipoylation levels↓
Gender		
[58]	Offspring of C57BL6/J female mice fed with a HFD which were separated by sex	Maternal HFD affected male offspring: inflammation↑ abnormal oxidative phosphorylation↑
Hormones		
[59]	Male mice were divided into control and glucocorticoid-treated groups	Glucocorticoids: BTG1-dependent autophagy↑
[60]	Male rabbits were divided into control and glucocorticoid-treated groups	Glucocorticoids: leptin↑ leptin receptor↓ mitochondrial markers↓ ATP content↓
[61]	Male Wistar rats were divided into control and glucocorticoid-treated groups	Glucocorticoids: miR-21-5p↑ mitochondrial fission/fusion/mitophagy genes↑
[62]	Female Sprague-Dawley rats were divided into sham operation group, OVX or Estrogen treatment after OVX group	Estrogen: hypothalamic ceramide↓ ER stress↓
[64]	Drd2 floxed mice (control) and pituitary prolactin secreting cells-deficient Drd2 mice which were created by crossing with Prl-Cre mice	Prolactin: thermogenic genes↓ mitochondrial dysfunction↑ inflammation↑
Tissue Microenvironment		
[65]	VEGFA floxed mice (control) and adipose-deficient VEGFA mice which were created by crossing with aP2-Cre mice	VEGFA deficiency: capillaries↓ β-adrenergic signaling↓ mtROS↑ mitophagy↑
[66]	Neonatal C57BL/6 mice with oxygen-induced retinopathy were intravitreally injected with PBS or anti-VEGF164 antibody	Anti-VEGF antibodies: VEGF↓ lipid accumulation↑ vascular density↓ mitochondrial genes↓
Genetic Regulation		
[11]	ChREBP-β floxed mice (control) and brown adipose-deficient ChREBP-β mice which were created by crossing with Ucp1-Cre mice	ChREBP-β overexpression: mitochondrial fission/fusion↓ thermogenic capacity↓
[67]	TFEB floxed mice (control) and brown adipose-deficient TFEB mice which were created by crossing with Ucp1-Cre mice	TFEB deficiency: LC3B-II accumulation↑ autophagic flux↑ mitochondrial degradation↑
[68]	KDM5A floxed mice (control) and brown adipose-deficient Kdm5a mice which were created by crossing with Ucp1-Cre mice	KDM5A overexpression: H3K4me3 at brown adipocyte-specific gene promoters↓
[69]	TFAM floxed mice (control) and adipose-deficient TFAM mice which were created by crossing with Adipo-Cre mice	TFAM deficiency: mitochondrial electron transport↓ fatty acid oxidation↓ circulating fatty acids↑
[92]	ERRγ floxed mice (control) and adipose-deficient ERRγ mice which were created by crossing with Adipo-Cre mice	ERRγ deficiency: Ucp1 and Fabp3 promoter expression↓
[93]	FAM195A whole-body knockout C57BL/6J mice and wild-type littermate controls	FAM195A deficiency: branched-chain amino↓ fatty acid metabolism↓
[94]	ATGL floxed mice (control) and adipose-deficient ATGL mice which were created by crossing with aP2-Cre	ATGL deficiency: lipolysis↓ fatty acid release↓ Pparα activity↓Ucp1↓
[95]	Dicer floxed mice (control) and adipose-deficient Dicer mice which were created by crossing with Adipo-Cre mice or tamoxifen-induced aP2-Cre	Dicer deficiency: brown to white adipocyte differentiation↑Ucp1/Elovl3↓leptin↑
[97]	Ucp1 whole-body knockout C57BL/6J mice and wild-type littermate controls	Ucp1 deficiency: mitochondrial subunits↓ inflammation↑ ER stress↑ oxidative stress genes↑
[98]	APOO floxed mice (control) and adipose-deficient APOO mice which were created by crossing with Adipo-Cre mice	APOO deficiency: mitochondrial fatty acid oxidation↓ peroxisome function↓
[70]	Noggin floxed mice (control) and adipose-deficient Noggin mice which were created by crossing with Adipo-Cre mice	Noggin deficiency: differentiation↓ thermogenesis↓ lipid metabolism genes↓

Abbreviations: *Adipo* adiponectin, *APOO* apolipoprotein O, *ATP* adenosine triphosphate, *ATF4* activating transcription factor 4, *ATGL* adipose triglyceride lipase, *aP2* adipocyte protein 2 (also known as Fabp4), *BAT* brown adipose tissue, *BPS* bisphenol S, *BTG1* B-cell translocation gene 1, *CaMK* calmodulin-dependent protein kinase, *CD36* cluster of differentiation 36, *cGMP* cyclic guanosine monophosphate, *CHOP* C/EBP homologous protein, *ChREBP-β* carbohydrate-responsive element-binding protein beta, *Cidea* cell death-inducing DFFA-like effector a, *Cre* Cre recombinase, *CYP1A1* cytochrome P450 family 1 subfamily A member 1, *DGAT2* diacylglycerol O-acyltransferase 2, *Dicer* Dicer ribonuclease III, *Dio2* type 2 iodothyronine deiodinase, *Drd2* dopamine receptor D2, *Elovl3* ELOVL fatty acid elongase 3, *ER* endoplasmic reticulum, *ERRγ* estrogen-related receptor gamma, *Fabp3* fatty acid-binding protein 3, *FAM195A* family with sequence similarity 195 member A, *FASN* fatty acid synthase, *Gadd45* growth arrest and DNA damage-inducible 45, *GR* glucocorticoid receptor, *GPR81* G protein-coupled receptor 81, *H3K4me3* histone H3 lysine 4 trimethylation, *HCD* high-carbohydrate diet, *HFD* high-fat diet, *HFrD* high-fructose diet, *HFSD* high-fat/sucrose diet, *IFN-γ* interferon-gamma, *IPO* interesterified palm oil, *KDM5A* lysine demethylase 5A, *LC3B-II* microtubule-associated protein phosphatidylethanolamine 1A/1B-light conjugate, *LETO* chain 3 Long-Evans Tokushima Otsuka (rat strain), *MAPK* mitogen-activated protein kinase, *miR-21-5p* microRNA-21-5p, *mtDNA* mitochondrial DNA, *mtROS* mitochondrial reactive oxygen species, *NLRP3* NLR family pyrin domain containing 3, *OLETF* Otsuka Long-Evans Tokushima Fatty (rat strain), *OVX* ovariectomy, *PBS* phosphate-buffered saline, *PGC-1α* peroxisome proliferator-activated receptor gamma coactivator 1-alpha, *PKG* protein kinase G, *PLIN1* perilipin 1, *PM2.5* fine particulate matter, *PO* palm oil, *PPAR* peroxisome proliferator-activated receptor, *PPARα* peroxisome proliferator-activated receptor alpha, *Prl* prolactin, *PSNPs* polystyrene nanoplastics, *Ras* rat sarcoma virus, *SREBP-1c* sterol regulatory element-binding protein 1c, *TFAM* mitochondrial transcription factor A, *TFEB* transcription factor EB, *TLR4* toll-like receptor 4, *UCP1* uncoupling protein 1, *VEGFA* vascular endothelial growth factor A, *Wnt* Wingless/Integrated, *n-3 FAs* omega-3 fatty acids.

Moreover, through an integrated approach combining literature review and computational bioinformatics, 4-HBA was identified as a candidate compound for BAT modulation. Our computational predictions showed partial alignment with a recent study reporting AMPK-DRP1 pathway activation by 4-HBA [18], suggesting possible mechanistic relevance that warrants experimental confirmation. We recognize that the observed interaction profile of 4-HBA may partly reflect target selection bias, as our protein panel was deliberately constructed around functionally validated regulators, enzymes, and effectors of BAT whitening. All targets were treated with equal weight to assess pathway-centric engagement rather than target prioritization, and redundancy or pathway overlap was retained to capture the multi-layered regulation of BAT whitening. This design enables assessment of broad engagement within this functionally defined network but does not permit conclusions regarding global binding selectivity. Accordingly, the finding that 4-HBA interacts with all 11 selected targets should be interpreted as evidence of pathway-wide engagement within this defined biological context. More quantitative, network-based approaches could further refine target prioritization in future studies.

Several critical limitations constrain the interpretative scope of these findings:

### **Methodological constraints**:

Despite its utility in initial screening, molecular docking has inherent limitations: its static modeling and predefined binding thresholds may not fully capture biological complexity. Consequently, these findings should be supplemented with dynamic simulations and experimental mutagenesis for robust validation.

### Polypharmacological risks:

The multi-target activity of the compound raises concerns about potential off-target interactions and tissue-specific toxicity, particularly through its engagement with mitochondrial and nuclear receptors. This complexity is further heightened by its metabolic conversion into active derivatives, which may exhibit distinct biological profiles.

### Clinical relevance gaps:

Although studies in rodent models provide foundational insights, the translational relevance of targeting BAT whitening, and specifically of the candidate metabolite 4-HBA, must be contextualized within key limitations. First, direct extrapolation to humans is constrained by interspecies variations in adipose tissue biology, depot distribution, and gut microbiota composition. Second, and more critically, no human pharmacokinetic or chronic safety data exist to inform the therapeutic dosing and risk profile of 4-HBA.

Therefore, while BAT modulation represents a theoretically promising strategy against metabolic disorders, the proposed role of 4-HBA remains exploratory and mechanistically suggestive. The integrated framework presented herein demonstrates a systematic strategy for prioritizing metabolites from bench to bedside. To bridge the current translational gap, future work must prioritize: (1) biophysical validation of metabolite-target interactions through structural biology approaches; (2) comparisons of 4-HBA efficacy across human and murine systems to elucidate translational potential; (3) multi-omics characterization of metabolite crosstalk across metabolic tissues to address interspecies physiological differences.

## Methods

### Literature Reviews

This review employed a structured narrative approach to synthesize evidence on BAT whitening. To ensure comprehensive and transparent literature coverage, a literature search was conducted across PubMed and Web of Science (2000–2025) using tailored Boolean operators (e.g., (brown adipose tissue OR BAT OR brown fat) AND (whiten* OR plastic* OR phenotypic switch)). All identified records were collated, deduplicated, and screened by title/abstract for relevance, followed by full-text assessment against predefined inclusion criteria: (i) original research or review articles; (ii) studies reporting key drivers, regulators, or determinants of BAT plasticity at the molecular, cellular, or physiological level; (iii) human and other mammalian models; (iv) English language publications.

Figures 1 and 2 were generated using BioRender.com, and no generative AI was involved in the figure design or creation process.

### Protein Panel Selection

A functional classification framework relevant to brown fat whitening was constructed by integrating function and compartment-based annotations from the Human Protein Atlas (v18.0) [123, 124] with the genome-scale functional annotation strategy [125]. Three categories were predefined—transcriptional/epigenetic regulators, metabolic enzymes, and transporters and secreted proteins—and within each category, candidate proteins were retained based on two sequential criteria: (i) documented functional association with brown fat whitening, especially concerning mitochondrial regulation, and (ii) availability of a high-resolution human crystal structure in UniProt to support downstream structural modeling. This selection process yielded 11 proteins: six transcriptional/epigenetic regulators—MLXIPL, TFEB, TFAM (transcription factors), ERRγ (nuclear receptor), KDM5A (histone demethylase), and FAM195A (RNA-binding protein); two metabolic enzymes—ATGL (lipase) and DICER (ribonuclease); and three transporters and secreted proteins—UCP1 (mitochondrial transporter), APOO (mitochondrial structural and secreted protein), and Noggin (secreted protein). All functional annotations for the selected proteins were retrieved from the UniProt Knowledgebase [126].

### Bioinformatics Analyses

Based on the protein panel selection, eleven proteins critically associated with adipose tissue plasticity and metabolic reprogramming (MLXIPL, TFEB, TFAM, ERRγ, KDM5A, FAM195A, ATGL, DICER, UCP1, APOO and Noggin) were selected as targets. Their three-dimensional structures were obtained from the UniProt database and prepared using AutoDockTools. Molecular docking-based virtual screening against the U.S. FDA-approved compound library was performed using AutoDock Vina. Docking poses were visualized and analyzed with PyMOL. Applying a binding energy threshold of ≤ − 5 kcal/mol, 4-HBA was identified as the sole compound demonstrating significant binding affinity across all eleven targets.

## Key References

- Peng Y, Zhao L, Li M, Liu Y, Shi Y, Zhang J : Plasticity of Adipose Tissues : Interconversion among White, Brown, and Beige Fat and Its Role in Energy Homeostasis. Biomolecules 2024, 14(4).
  - ○ This study provides a comprehensive overview of adipose tissue metabolic plasticity, encompassing phenomena such as BAT whitening, and highlights the therapeutic potential of targeting these processes for obesity treatment.
- Kim SH, Park WY, Song G, Park JY, Jung SJ, Ahn KS, et al : 4-hydroxybenzoic acid induces browning of white adipose tissue through the AMPK-DRP1 pathway in HFD-induced obese mice. Phytomedicine 2025, 137 :156353.
  - ○ This study provides direct *in vivo* evidence that 4-Hydroxybenzoic acid combats high-fat-diet-induced obesity by activating adipose thermogenesis.
- Mori MP, Lozoya OA, Santos JH: BAT mitochondria: whitening meets softening. Nat Metab 2025, 7(8):1503-1504.
  - ○ This study provides key evidence that mitochondrial stress and dysfunction drive BAT whitening through D-2HG-mediated epigenetic and nuclear mechanical remodeling, which offers crucial support for the main mechanism in this review.
- UniProt C : UniProt: the Universal Protein Knowledgebase in 2025. Nucleic Acids Res 2025, 53(D1): D609-D617.
  - ○ This study provides the essential reference for the UniProt database, which supplied both the crystal structure and functional annotations of protein.

## Data Availability

All data generated or analyzed during this study are included in this published article.

## References

[1] Yanovski SZ, Yanovski JA. Approach to obesity treatment in primary care: a review. JAMA Intern Med. 2024;184(7):818–29. 38466272 10.1001/jamainternmed.2023.8526 PMC12182808

[2] Zhang X, Liu J, Ni Y, Yi C, Fang Y, Ning Q, et al. Global prevalence of overweight and obesity in children and adolescents: a systematic review and meta-analysis. JAMA Pediatr. 2024;178(8):800–13. 38856986 10.1001/jamapediatrics.2024.1576 PMC11165417

[3] An SM, Cho SH, Yoon JC. Adipose Tissue and Metabolic Health. Diabetes Metab J. 2023;47(5):595–611. 37482656 10.4093/dmj.2023.0011 PMC10555533

[4] Karanfil AS, Louis F, Matsusaki M. Brown adipose tissue engineering: advances, challenges, and future directions. Trends Biotechnol; 2025. 10.1016/j.tibtech.2025.08.010 41006179

[5] Cypess AM, Cannon B, Nedergaard J, Kazak L, Chang DC, Krakoff J, et al. Emerging debates and resolutions in brown adipose tissue research. Cell Metab. 2025;37(1):12–33. 39644896 10.1016/j.cmet.2024.11.002 PMC11710994

[6] Zhao L, Zhu QJ, Li MH, Yang HL, Zhao YJ. Revisiting brown adipose tissue whitening: physiological mechanisms and pathophysiological consequences. J Physiol. 2025;603(19):5299–326. 40930151 10.1113/JP289263

[7] Ziqubu K, Dludla PV, Mthembu SXH, Nkambule BB, Mabhida SE, Jack BU, et al. An insight into brown/beige adipose tissue whitening, a metabolic complication of obesity with the multifactorial origin. Front Endocrinol (Lausanne). 2023;14:1114767. 36875450 10.3389/fendo.2023.1114767 PMC9978510

[8] Sakers A, De Siqueira MK, Seale P, Villanueva CJ. Adipose-tissue plasticity in health and disease. Cell. 2022;185(3):419–46. 35120662 10.1016/j.cell.2021.12.016 PMC11152570

[9] Kotzbeck P, Giordano A, Mondini E, Murano I, Severi I, Venema W, et al. Brown adipose tissue whitening leads to brown adipocyte death and adipose tissue inflammation. J Lipid Res. 2018;59(5):784–94. 29599420 10.1194/jlr.M079665 PMC5928436

[10] Glauser JSO, Santana-Oliveira DA, Silva-Veiga FM, Fernandes-da-Silva A, Aguila MB, Souza-Mello V. Excessive dietary saturated fat or fructose and their combination (found in ultra-processed foods) impair mitochondrial dynamics markers and cause brown adipocyte whitening in adult mice. Nutrition. 2025;137:112805. 40378644 10.1016/j.nut.2025.112805

[11] Wei C, Ma X, Su K, Qi S, Zhu Y, Lin J, Wang C, Yang R, Chen X, Wang W, et al. ChREBP-beta regulates thermogenesis in brown adipose tissue. J Endocrinol. 2020;245(3):343–56. 32208359 10.1530/JOE-19-0498

[12] Peng Y, Zhao L, Li M, Liu Y, Shi Y, Zhang J. Plasticity of Adipose Tissues: interconversion among White, Brown, and Beige Fat and its role in energy homeostasis. Biomolecules. 2024;14(4):483.. 38672499 10.3390/biom14040483 PMC11048349

[13] Cairo M, Villarroya J. The role of autophagy in brown and beige adipose tissue plasticity. J Physiol Biochem. 2020;76(2):213–26. 31811543 10.1007/s13105-019-00708-1

[14] Cui X, Nguyen NL, Zarebidaki E, Cao Q, Li F, Zha L, et al. Thermoneutrality decreases thermogenic program and promotes adiposity in high-fat diet-fed mice. Physiol Rep. 2016;4(10):e12799. 27230905 10.14814/phy2.12799 PMC4886167

[15] Miranda CS, Silva-Veiga F, Martins FF, Rachid TL, Mandarim-De-Lacerda CA, Souza-Mello V. PPAR-alpha activation counters brown adipose tissue whitening: a comparative study between high-fat- and high-fructose-fed mice. Nutrition. 2020;78:110791. 32682271 10.1016/j.nut.2020.110791

[16] Li L, Wan Q, Long Q, Nie T, Zhao S, Mao L, et al. Comparative transcriptomic analysis of rabbit interscapular brown adipose tissue whitening under physiological conditions. Adipocyte. 2022;11(1):529–49. 36000239 10.1080/21623945.2022.2111053 PMC9427046

[17] Wang X, Qi Y, Zheng H. Dietary polyphenol, gut microbiota, and health benefits. Antioxidants (Basel). 2022;11(6):1212. 35740109 10.3390/antiox11061212 PMC9220293

[18] Kim SH, Park WY, Song G, Park JY, Jung SJ, Ahn KS, et al. 4-hydroxybenzoic acid induces browning of white adipose tissue through the AMPK-DRP1 pathway in HFD-induced obese mice. Phytomedicine. 2025;137:156353. 39799892 10.1016/j.phymed.2024.156353

[19] Lee P, Bova R, Schofield L, Bryant W, Dieckmann W, Slattery A, et al. Brown adipose tissue exhibits a glucose-responsive thermogenic biorhythm in humans. Cell Metab. 2016;23(4):602–9. 26972823 10.1016/j.cmet.2016.02.007

[20] Negroiu CE, Tudorascu I, Bezna CM, Godeanu S, Diaconu M, Danoiu R, et al. Beyond the cold: activating brown adipose tissue as an approach to combat obesity. J Clin Med. 2024;13(7):1973. 38610736 10.3390/jcm13071973 PMC11012454

[21] Carpentier AC, Blondin DP, Haman F, Richard D. Brown adipose tissue-a translational perspective. Endocr Rev. 2023;44(2):143–92. 35640259 10.1210/endrev/bnac015 PMC9985413

[22] Seki T, Yang Y, Sun X, Lim S, Xie S, Guo Z, et al. Brown-fat-mediated tumour suppression by cold-altered global metabolism. Nature. 2022;608(7922):421–8. 35922508 10.1038/s41586-022-05030-3 PMC9365697

[23] Ghesmati Z, Rashid M, Fayezi S, Gieseler F, Alizadeh E, Darabi M. An update on the secretory functions of brown, white, and beige adipose tissue: towards therapeutic applications. Rev Endocr Metab Disord. 2023. 10.1007/s11154-023-09850-0. 38051471 10.1007/s11154-023-09850-0 PMC10942928

[24] Wibmer AG, Becher T, Eljalby M, Crane A, Andrieu PC, Jiang CS, et al. Brown adipose tissue is associated with healthier body fat distribution and metabolic benefits independent of regional adiposity. Cell Rep Med. 2021;2(7):100332. 34337558 10.1016/j.xcrm.2021.100332 PMC8324464

[25] Becher T, Palanisamy S, Kramer DJ, Eljalby M, Marx SJ, Wibmer AG, et al. Brown adipose tissue is associated with cardiometabolic health. Nat Med. 2021;27(1):58–65. 33398160 10.1038/s41591-020-1126-7 PMC8461455

[26] Wu J, Bostrom P, Sparks LM, Ye L, Choi JH, Giang AH, et al. Beige adipocytes are a distinct type of thermogenic fat cell in mouse and human. Cell. 2012;150(2):366–76. 22796012 10.1016/j.cell.2012.05.016 PMC3402601

[27] Sharp LZ, Shinoda K, Ohno H, Scheel DW, Tomoda E, Ruiz L, et al. Human BAT possesses molecular signatures that resemble beige/brite cells. PLoS One. 2012;7(11):e49452. 23166672 10.1371/journal.pone.0049452 PMC3500293

[28] de Jong JMA, Sun W, Pires ND, Frontini A, Balaz M, Jespersen NZ, et al. Human brown adipose tissue is phenocopied by classical brown adipose tissue in physiologically humanized mice. Nat Metab. 2019;1(8):830–43. 32694768 10.1038/s42255-019-0101-4

[29] de Jong JMA, Cannon B, Nedergaard J, Wolfrum C, Petrovic N. Reply to “Confounding issues in the ‘humanized’ brown fat of mice.” Nat Metab. 2020;2(4):305–6. 32694605 10.1038/s42255-020-0193-x

[30] Cypess AM, White AP, Vernochet C, Schulz TJ, Xue R, Sass CA, et al. Anatomical localization, gene expression profiling and functional characterization of adult human neck brown fat. Nat Med. 2013;19(5):635–9. 23603815 10.1038/nm.3112 PMC3650129

[31] Cannon B, de Jong JMA, Fischer AW, Nedergaard J, Petrovic N. Human brown adipose tissue: classical brown rather than brite/beige? Exp Physiol. 2020;105(8):1191–200. 32378255 10.1113/EP087875

[32] Lidell ME, Betz MJ, Dahlqvist Leinhard O, Heglind M, Elander L, Slawik M, et al. Evidence for two types of brown adipose tissue in humans. Nat Med. 2013;19(5):631–4. 23603813 10.1038/nm.3017

[33] Kaikaew K, Grefhorst A, Visser JA. Sex differences in brown adipose tissue function: sex hormones, glucocorticoids, and their crosstalk. Front Endocrinol (Lausanne). 2021;12:652444. 33927694 10.3389/fendo.2021.652444 PMC8078866

[34] Fletcher LA, Kim K, Leitner BP, Cassimatis TM, O’Mara AE, Johnson JW, Halprin MS, McGehee SM, Brychta RJ, Cypess AM, et al. Sexual Dimorphisms in Adult Human Brown Adipose Tissue. Obes (Silver Spring). 2020;28(2):241–6. 10.1002/oby.22698 PMC6986330 31970907

[35] Greenhill C. Obesity: cold exposure increases brown adipose tissue in humans. Nat Rev Endocrinol. 2013;9(10):566. 23917582 10.1038/nrendo.2013.156

[36] Cai Z, Zhong Q, Feng Y, Wang Q, Zhang Z, Wei C, et al. Non-invasive mapping of brown adipose tissue activity with magnetic resonance imaging. Nat Metab. 2024;6(7):1367–79. 39054361 10.1038/s42255-024-01082-z PMC11272596

[37] Straat ME, Hoekx CA, van Velden FHP, Pereira Arias-Bouda LM, Dumont L, Blondin DP, et al. Stimulation of the beta-2-adrenergic receptor with salbutamol activates human brown adipose tissue. Cell Rep Med. 2023;4(2):100942. 36812890 10.1016/j.xcrm.2023.100942 PMC9975328

[38] Dabrowska AM, Dudka J. Mirabegron, a selective beta3-adrenergic receptor agonist, as a potential anti-obesity drug. J Clin Med. 2023;12(21):6897. 10.3390/jcm12216897 PMC10649615 37959362

[39] Nahon KJ, Janssen LGM, Sardjoe Mishre ASD, Bilsen MP, van der Eijk JA, Botani K, et al. The effect of mirabegron on energy expenditure and brown adipose tissue in healthy lean South Asian and Europid men. Diabetes Obes Metab. 2020;22(11):2032–44. 32558052 10.1111/dom.14120 PMC7771034

[40] Bittencourt JOA, Marcondes-de-Castro IA, Marinho TS, Aguila MB, Mandarim-de-Lacerda CA. Tirzepatide counteracts brown adipose tissue whitening, inflammation, and mitochondrial dysfunction in estrogen-deficient obese diabetic mice. Life Sci. 2025;386:124155. 41412277 10.1016/j.lfs.2025.124155

[41] de Oliveira Silva T, Lunardon G, Lino CA, de Almeida Silva A, Zhang S, Irigoyen MCC, et al. Senescent cell depletion alleviates obesity-related metabolic and cardiac disorders. Mol Metab. 2025;91:102065. 39557194 10.1016/j.molmet.2024.102065 PMC11636344

[42] Awazawa M, Matsushita M, Nomura I, Kobayashi N, Tamura-Nakano M, Sorimachi Y, et al. Imeglimin improves systemic metabolism by targeting brown adipose tissue and gut microbiota in obese model mice. Metabolism. 2024;153:155796. 38262576 10.1016/j.metabol.2024.155796

[43] Tanaka Y, Nagoshi T, Takahashi H, Oi Y, Yoshii A, Kimura H, et al. URAT1-selective inhibition ameliorates insulin resistance by attenuating diet-induced hepatic steatosis and brown adipose tissue whitening in mice. Mol Metab. 2022;55:101411. 34863940 10.1016/j.molmet.2021.101411 PMC8717577

[44] Huang W, Zhu W, Lin Y, Chan FKL, Xu Z, Ng SC. *Roseburia hominis* improves host metabolism in diet-induced obesity. Gut Microbes. 2025;17(1):2467193. 39976263 10.1080/19490976.2025.2467193 PMC11845086

[45] Hao L, Khan MSH, Zu Y, Liu J, Wang S. Thermoneutrality inhibits thermogenic markers and exacerbates nonalcoholic fatty liver disease in mice. Int J Mol Sci. 2024;25(15):8482. 39126051 10.3390/ijms25158482 PMC11312964

[46] Fruhbeck G, Mendez-Gimenez L, Becerril S, Ramirez B, Hernandez-Pardos AW, Cienfuegos JA, et al. Increased aquaporin-7 expression is associated with changes in rat brown adipose tissue whitening in obesity: impact of cold exposure and bariatric surgery. Int J Mol Sci. 2023;24(4):3412. 36834823 10.3390/ijms24043412 PMC9963055

[47] Gao M, Ma Y, Liu D. High-fat diet-induced adiposity, adipose inflammation, hepatic steatosis and hyperinsulinemia in outbred CD-1 mice. PLoS One. 2015;10(3):e0119784. 25768847 10.1371/journal.pone.0119784 PMC4358885

[48] Rangel-Azevedo C, Santana-Oliveira DA, Miranda CS, Martins FF, Mandarim-de-Lacerda CA, Souza-Mello V. Progressive brown adipocyte dysfunction: whitening and impaired nonshivering thermogenesis as long-term obesity complications. J Nutr Biochem. 2022;105:109002. 35346828 10.1016/j.jnutbio.2022.109002

[49] Kuipers EN, Held NM, In Het Panhuis W, Modder M, Ruppert PMM, Kersten S, et al. A single day of high-fat diet feeding induces lipid accumulation and insulin resistance in brown adipose tissue in mice. Am J Physiol Endocrinol Metab. 2019;317(5):E820–30. 31386566 10.1152/ajpendo.00123.2019

[50] Della Guardia L, Shin AC. White and brown adipose tissue functionality is impaired by fine particulate matter (PM(2.5)) exposure. J Mol Med (Berl). 2022;100(5):665–76. 35286401 10.1007/s00109-022-02183-6 PMC9110515

[51] Peshdary V, Styles G, Rigden M, Caldwell D, Kawata A, Sorisky A, et al. Exposure to low doses of Dechlorane Plus promotes adipose tissue dysfunction and glucose intolerance in male mice. Endocrinology. 2020;161(8):bqaa096. 32556108 10.1210/endocr/bqaa096

[52] Fan J, Ping J, Zhang WX, Rao YS, Liu HX, Zhang J, Yan YE. Prenatal and lactation nicotine exposure affects morphology and function of brown adipose tissue in male rat offspring. Ultrastruct Pathol. 2016;40(5):288–95. 27598972 10.1080/01913123.2016.1223243

[53] Nguyen ST, Fujita N, Oshima T, Nishihira M, Ohno H, Yoneda M, Urakawa S. Effects of long-term childhood exercise and detraining on lipid accumulation in metabolic-related organs. PLoS ONE. 2022;17(6):e0270330. 35749411 10.1371/journal.pone.0270330 PMC9231767

[54] Takaishi K, Oshima T, Eto H, Nishihira M, Nguyen ST, Ochi R, et al. Impact of exercise and detraining during childhood on brown adipose tissue whitening in obesity. Metabolites. 2021;11(10):677. 34677392 10.3390/metabo11100677 PMC8540482

[55] Yao Z, Liang S, Chen J, Zhang H, Chen W, Li H. Dietary lactate intake and physical exercise synergistically reverse brown adipose tissue whitening to ameliorate diet-induced obesity. J Agric Food Chem. 2024;72(45):25286–297. 39486070 10.1021/acs.jafc.4c06899

[56] Nguyen TT, Corvera S. Adipose tissue as a linchpin of organismal ageing. Nat Metab. 2024;6(5):793–807. 38783156 10.1038/s42255-024-01046-3 PMC11238912

[57] Goncalves LF, Machado TQ, Castro-Pinheiro C, de Souza NG, Oliveira KJ, Fernandes-Santos C. Ageing is associated with brown adipose tissue remodelling and loss of white fat browning in female C57BL/6 mice. Int J Exp Pathol. 2017;98(2):100–8. 28543963 10.1111/iep.12228 PMC5485357

[58] Savva C, Helguero LA, Gonzalez-Granillo M, Melo T, Couto D, Buyandelger B, et al. Maternal high-fat diet programs white and brown adipose tissue lipidome and transcriptome in offspring in a sex- and tissue-dependent manner in mice. Int J Obes (Lond). 2022;46(4):831–42. 34997206 10.1038/s41366-021-01060-5 PMC8960419

[59] Deng J, Guo Y, Yuan F, Chen S, Yin H, Jiang X, et al. Autophagy inhibition prevents glucocorticoid-increased adiposity via suppressing BAT whitening. Autophagy. 2020;16(3):451–65. 31184563 10.1080/15548627.2019.1628537 PMC6999619

[60] Tang X, Zhang B, Xie P, Wei Y, Qiu Y, Yi X, et al. Dexamethasone-induced whitening of rabbit brown adipose tissue: leptin resistance and mitochondrial dysfunction. BMC Genomics. 2025;26(1):326. 40165063 10.1186/s12864-025-11502-3 PMC11959718

[61] Bolin AP, de Fatima Silva F, Salgueiro RB, Dos Santos BA, Komino ACM, Andreotti S, et al. Glucocorticoid modulates oxidative and thermogenic function of rat brown adipose tissue and human brown adipocytes. J Cell Physiol. 2024;239(9):1–12. 39091018 10.1002/jcp.31397 PMC12617538

[62] Gonzalez-Garcia I, Contreras C, Estevez-Salguero A, Ruiz-Pino F, Colsh B, Pensado I, et al. Estradiol regulates energy balance by ameliorating hypothalamic ceramide-induced ER stress. Cell Rep. 2018;25(2):413–423.e415. 30304681 10.1016/j.celrep.2018.09.038 PMC6198289

[63] Gasparini SJ, Swarbrick MM, Kim S, Thai LJ, Henneicke H, Cavanagh LL, Tu J, Weber MC, Zhou H, Seibel MJ. Androgens sensitise mice to glucocorticoid-induced insulin resistance and fat accumulation. Diabetologia. 2019;62(8):1463–77. 31098671 10.1007/s00125-019-4887-0

[64] Lopez-Vicchi F, De Winne C, Ornstein AM, Sorianello E, Toneatto J, Becu-Villalobos D. Severe hyperprolactinemia promotes brown adipose tissue whitening and aggravates high fat diet induced metabolic imbalance. Front Endocrinol (Lausanne). 2022;13:883092. 35757410 10.3389/fendo.2022.883092 PMC9226672

[65] Shimizu I, Aprahamian T, Kikuchi R, Shimizu A, Papanicolaou KN, MacLauchlan S, Maruyama S, Walsh K. Vascular rarefaction mediates whitening of brown fat in obesity. J Clin Invest. 2014;124(5):2099–112. 24713652 10.1172/JCI71643 PMC4001539

[66] Jo DH, Park SW, Cho CS, Powner MB, Kim JH, Fruttiger M, et al. Intravitreally injected anti-VEGF antibody reduces brown fat in neonatal mice. PLoS ONE. 2015;10(7):e0134308. 26226015 10.1371/journal.pone.0134308 PMC4520452

[67] Sass F, Schlein C, Jaeckstein MY, Pertzborn P, Schweizer M, Schinke T, et al. TFEB deficiency attenuates mitochondrial degradation upon brown adipose tissue whitening at thermoneutrality. Mol Metab. 2021;47:101173. 33516944 10.1016/j.molmet.2021.101173 PMC7903014

[68] Wen X, Xiao Y, Xiao H, Tan X, Wu B, Li Z, et al. Bisphenol S induces brown adipose tissue whitening and aggravates diet-induced obesity in an estrogen-dependent manner. Cell Rep. 2023;42(12):113504. 38041811 10.1016/j.celrep.2023.113504

[69] Vernochet C, Damilano F, Mourier A, Bezy O, Mori MA, Smyth G, Rosenzweig A, Larsson NG, Kahn CR. Adipose tissue mitochondrial dysfunction triggers a lipodystrophic syndrome with insulin resistance, hepatosteatosis, and cardiovascular complications. FASEB J. 2014;28(10):4408–19. 25005176 10.1096/fj.14-253971 PMC4202105

[70] Blazquez-Medela AM, Jumabay M, Rajbhandari P, Sallam T, Guo Y, Yao J, et al. Noggin depletion in adipocytes promotes obesity in mice. Mol Metab. 2019;25:50–63. 31027994 10.1016/j.molmet.2019.04.004 PMC6600080

[71] Johnson F, Mavrogianni A, Ucci M, Vidal-Puig A, Wardle J. Could increased time spent in a thermal comfort zone contribute to population increases in obesity? Obes Rev. 2011;12(7):543–51. 21261804 10.1111/j.1467-789X.2010.00851.x

[72] Yoneshiro T, Matsushita M, Nakae S, Kameya T, Sugie H, Tanaka S, Saito M. Brown adipose tissue is involved in the seasonal variation of cold-induced thermogenesis in humans. Am J Physiol Regul Integr Comp Physiol. 2016;310(10):R999–1009. 27030666 10.1152/ajpregu.00057.2015

[73] Da Eira D, Jani S, Ceddia RB. An obesogenic diet impairs uncoupled substrate oxidation and promotes whitening of the brown adipose tissue in rats. J Physiol. 2023;601(1):69–82. 36419345 10.1113/JP283721

[74] Serdan TDA, Masi LN, Pereira JNB, Rodrigues LE, Alecrim AL, Scervino MVM, et al. Impaired brown adipose tissue is differentially modulated in insulin-resistant obese wistar and type 2 diabetic Goto-Kakizaki rats. Biomed Pharmacother. 2021;142:112019. 34403962 10.1016/j.biopha.2021.112019

[75] Martins BC, Soares AC, Martins FF, Resende AC, Inada KOP, Souza-Mello V, et al. Coffee consumption prevents obesity-related comorbidities and attenuates brown adipose tissue whitening in high-fat diet-fed mice. J Nutr Biochem. 2023;117:109336. 36990367 10.1016/j.jnutbio.2023.109336

[76] Bellitto V, Gabrielli MG, Martinelli I, Roy P, Nittari G, Cocci P, et al. Dysfunction of the brown adipose organ in HFD-obese rats and effect of tart cherry supplementation. Antioxidants (Basel). 2024;13(4):388. 38671836 10.3390/antiox13040388 PMC11047636

[77] Saban Guler M, Yildiran H, Seymen CM. Effect of omega-3 fatty acids on adipose tissue: histological, metabolic, and gene expression analyses in mice fed a high-fat diet. Sci Rep. 2025;15(1):37179. 41131120 10.1038/s41598-025-22396-2 PMC12549936

[78] Peixoto TC, Quitete FT, Teixeira AVS, Martins BC, Soares RA, Atella GC, et al. Palm and interesterified palm oil-enhanced brown fat whitening contributes to metabolic dysfunction in C57BL/6J mice. Nutr Res. 2025;133:94–107. 39705913 10.1016/j.nutres.2024.11.009

[79] Song SL, Chen XY, Zhao J, Li YY, Xiong YM, Lv L, et al. Effects of the fungicide Prothioconazole on lipid metabolism in mice: whitening alterations of brown adipose tissue. Environ Sci Technol. 2024;58(41):18155–66. 39361549 10.1021/acs.est.4c05666

[80] Shen Z, Tian K, Tang J, Wang L, Zhang F, Yang L, et al. Exposure to nanoplastics during pregnancy induces brown adipose tissue whitening in male offspring. Toxics. 2025;13(3):171. 40137498 10.3390/toxics13030171 PMC11945425

[81] Jiang X, Li M, Li W, Guo Y, Zhang J, Ye L, et al. Effects of co-exposure to heat and ozone on lipid metabolism in the liver and adipose tissue of C57BL/6J male mice. J Hazard Mater. 2025;489:137577. 39947076 10.1016/j.jhazmat.2025.137577

[82] Virtanen KA. BAT thermogenesis: linking shivering to exercise. Cell Metab. 2014;19(3):352–4. 24606895 10.1016/j.cmet.2014.02.013

[83] Bao JF, She QY, Hu PP, Jia N, Li A. Irisin, a fascinating field in our times. Trends Endocrinol Metab. 2022;33(9):601–13. 35872067 10.1016/j.tem.2022.06.003

[84] Nirengi S, Stanford K. Brown adipose tissue and aging: a potential role for exercise. Exp Gerontol. 2023;178:112218. 37224933 10.1016/j.exger.2023.112218 PMC12150252

[85] Du K, Bai X, Yang L, Shi Y, Chen L, Wang H, et al. De novo reconstruction of transcriptome identified long non-coding RNA regulator of aging-related brown adipose tissue whitening in rabbits. Biology. 2021;10(11):1176. 34827171 10.3390/biology10111176 PMC8614855

[86] Du K, Bai X, Chen L, Shi Y, Wang HD, Cai MC, et al. Integrated analysis of microRNAs, circular RNAs, long non-coding RNAs, and mRNAs revealed competing endogenous RNA networks involved in brown adipose tissue whitening in rabbits. BMC Genomics. 2022;23(1):779. 36443655 10.1186/s12864-022-09025-2 PMC9703717

[87] Huang Z, Zhang Z, Moazzami Z, Heck R, Hu P, Nanda H, et al. Brown adipose tissue involution associated with progressive restriction in progenitor competence. Cell Rep. 2022;39(2):110575. 35417710 10.1016/j.celrep.2022.110575 PMC9664906

[88] Grana-Baumgartner A, Dukkipati VSR, Kenyon PR, Blair HT, Lopez-Villalobos N, Gedye K, et al. RNAseq analysis of brown adipose tissue and thyroid of newborn lambs subjected to short-term cold exposure reveals signs of early whitening of adipose tissue. Metabolites. 2022;12(10):996. 36295898 10.3390/metabo12100996 PMC9607389

[89] Pan XX, Yao KL, Yang YF, Ge Q, Zhang R, Gao PJ, et al. Senescent T cell induces brown adipose tissue “whitening” via secreting IFN-gamma. Front Cell Dev Biol. 2021;9:637424. 33748126 10.3389/fcell.2021.637424 PMC7969812

[90] Tajima K, Ikeda K, Chang HY, Chang CH, Yoneshiro T, Oguri Y, Jun H, Wu J, Ishihama Y, Kajimura S. Mitochondrial lipoylation integrates age-associated decline in brown fat thermogenesis. Nat Metab. 2019;1(9):886–98. 32313871 10.1038/s42255-019-0106-z PMC7169975

[91] Pfannenberg C, Werner MK, Ripkens S, Stef I, Deckert A, Schmadl M, Reimold M, Haring HU, Claussen CD, Stefan N. Impact of age on the relationships of brown adipose tissue with sex and adiposity in humans. Diabetes. 2010;59(7):1789–93. 20357363 10.2337/db10-0004 PMC2889780

[92] Ahmadian M, Liu S, Reilly SM, Hah N, Fan W, Yoshihara E, Jha P, De Magalhaes Filho CD, Jacinto S, Gomez AV, et al. ERRgamma Preserves Brown Fat Innate Thermogenic Activity. Cell Rep. 2018;22(11):2849–59. 29539415 10.1016/j.celrep.2018.02.061 PMC5884669

[93] Cannavino J, Shao M, An YA, Bezprozvannaya S, Chen S, Kim J, et al. Regulation of cold-induced thermogenesis by the RNA binding protein FAM195A. Proc Natl Acad Sci U S A. 2021. 10.1073/pnas.2104650118. 34088848 10.1073/pnas.2104650118 PMC8201964

[94] Ahmadian M, Abbott MJ, Tang T, Hudak CS, Kim Y, Bruss M, et al. Desnutrin/ATGL is regulated by AMPK and is required for a brown adipose phenotype. Cell Metab. 2011;13(6):739–48. 21641555 10.1016/j.cmet.2011.05.002 PMC3148136

[95] Mori MA, Thomou T, Boucher J, Lee KY, Lallukka S, Kim JK, et al. Altered miRNA processing disrupts brown/white adipocyte determination and associates with lipodystrophy. J Clin Invest. 2014;124(8):3339–51. 24983316 10.1172/JCI73468 PMC4109560

[96] Winn NC, Grunewald ZI, Gastecki ML, Woodford ML, Welly RJ, Clookey SL, et al. Deletion of UCP1 enhances ex vivo aortic vasomotor function in female but not male mice despite similar susceptibility to metabolic dysfunction. Am J Physiol Endocrinol Metab. 2017;313(4):E402–12. 28655717 10.1152/ajpendo.00096.2017 PMC5668596

[97] Winn NC, Vieira-Potter VJ, Gastecki ML, Welly RJ, Scroggins RJ, Zidon TM, et al. Loss of UCP1 exacerbates Western diet-induced glycemic dysregulation independent of changes in body weight in female mice. Am J Physiol Regul Integr Comp Physiol. 2017;312(1):R74–84. 27881400 10.1152/ajpregu.00425.2016 PMC5283932

[98] Guo X, Hu J, He G, Chen J, Yang Y, Qin D, et al. Loss of APOO (MIC26) aggravates obesity-related whitening of brown adipose tissue via PPARalpha-mediated functional interplay between mitochondria and peroxisomes. Metabolism. 2023;144:155564. 37088120 10.1016/j.metabol.2023.155564

[99] Yuan N, Shen L, Peng Q, Sha R, Wang Z, Xie Z, et al. SRSF1 is required for mitochondrial homeostasis and thermogenic function in brown adipocytes through its control of Ndufs3 splicing. Adv Sci (Weinh). 2024;11(21):e2306871. 38569495 10.1002/advs.202306871 PMC11151030

[100] Wang H, Zhang L, Chen X, Hong L, Zhao J, Qian W, et al. Adipocyte-specific Steap4 deficiency reduced thermogenesis and energy expenditure in mice. iScience. 2025;28(2):111903. 39995871 10.1016/j.isci.2025.111903 PMC11848796

[101] Sun C, Liang J, Zheng J, Mao S, Chen S, Aikemu A, et al. Brown adipose Vanin-1 is required for the maintenance of mitochondrial homeostasis and prevents diet-induced metabolic dysfunction. Mol Metab. 2024;80:101884. 38246587 10.1016/j.molmet.2024.101884 PMC10838954

[102] Mori MP, Lozoya OA, Santos JH. BAT mitochondria: whitening meets softening. Nat Metab. 2025;7(8):1503–4. 40750943 10.1038/s42255-025-01319-5

[103] Santiago PJD, Shimada BK, Alfulaij N, Hallam KA, Soares AG, Young V, Swanson SM, Remedios GL, Toh P, Seale LA. Targeted disruption of selenocysteine lyase in brown adipocytes controls glutathione peroxidase 1 and 4 expression in males. Biol Trace Elem Res. 2025. 10.1007/s12011-025-04904-7 PMC13149581 41366172

[104] Castelli S, Tramutola A, Perluigi M, Bacalini MG, Ciriolo MR, Ciccarone F. Oxidative stress characterizes the dysfunction of thermogenic adipose tissue in a mouse model of down syndrome. Free Radic Biol Med. 2025;237:101–9. 40456497 10.1016/j.freeradbiomed.2025.05.432

[105] Kaul H, Isermann L, Senft K, Popovic M, Georgomanolis T, Baumann L, et al. 2-hydroxyglutarate mediates whitening of brown adipocytes coupled to nuclear softening upon mitochondrial dysfunction. Nat Metab. 2025;7(8):1593–613. 40750944 10.1038/s42255-025-01332-8 PMC12373511

[106] Favero G, Golic I, Arnaboldi F, Cappella A, Korac A, Monsalve M, et al. Cardiometabolic changes in Sirtuin1-heterozygous mice on high-fat diet and melatonin supplementation. Int J Mol Sci. 2024;25(2):860. 38255934 10.3390/ijms25020860 PMC10815439

[107] Wang S, He T, Luo Y, Ren K, Shen H, Hou L, et al. SOX4 facilitates brown fat development and maintenance through EBF2-mediated thermogenic gene program in mice. Cell Death Differ. 2025;32(3):447–65. 39402212 10.1038/s41418-024-01397-0 PMC11893884

[108] Lee SY, Fontana F, Sugatani T, Portales Castillo I, Leanza G, Coler-Reilly A, Civitelli R. Connexin43 in mesenchymal lineage cells regulates body adiposity and energy metabolism in mice. JCI Insight. 2024;9(6):e170016. 10.1172/jci.insight.170016 PMC11063945 38349739

[109] Nozaki Y, Kobayashi M, Fukuoh T, Ishimatsu M, Narita T, Taki K, et al. Mipep deficiency in adipocytes impairs mitochondrial protein maturation and leads to systemic inflammation and metabolic dysfunctions. Sci Rep. 2025;15(1):12839. 40229443 10.1038/s41598-025-97307-6 PMC11997187

[110] Zhou Q, Lu Z, Wang B, Wang Y, Li L, You M, et al. Endothelial SIRT3 deficiency predisposes brown adipose tissue to whitening in diet-induced obesity. Int J Biol Sci. 2025;21(8):3444–60. 40520025 10.7150/ijbs.110741 PMC12160546

[111] Urano Y, Mii S, Asai S, Esaki N, Ando R, Shiraki Y, et al. Superoxide dismutase 2 deficiency in mesenchymal stromal cells induces sympathetic denervation and functional impairment of brown adipose tissue. Pathol Int. 2025;75(2):69–81. 39760485 10.1111/pin.13503 PMC11848962

[112] Eberhardt J, Santos-Martins D, Tillack AF, Forli S. Autodock Vina 1.2.0: new docking methods, expanded force field, and Python bindings. J Chem Inf Model. 2021;61(8):3891–8. 34278794 10.1021/acs.jcim.1c00203 PMC10683950

[113] Wang Z, Zhang S, Li S, Zhao G, Zhang Y, Zhang X, Sun M, Lu Y, Song E, Quan C, et al. Mechanism by which CAP regulates the cold-heat balance in osteosarcoma model mice: an integrative study of metabolomics, transcriptomics, and network pharmacology. J Transl Med. 2025;23(1):1177. 41146125 10.1186/s12967-025-07238-z PMC12560403

[114] Cheng F, Gao H, Yan B, Chen F, Lei P. Triclosan exposure potentiates ischemic stroke risk: multi-omics integration and molecular docking unveil neurotoxic mechanisms. Ecotoxicol Environ Saf. 2025;302:118551. 40544545 10.1016/j.ecoenv.2025.118551

[115] Shamsol Azman ANS, Tan JJ, Abdullah MNH, Bahari H, Lim V, Yong YK. Network pharmacology and molecular docking analysis of active compounds in Tualang honey against atherosclerosis. Foods. Foods. 2023;12(9):1779. 37174317 10.3390/foods12091779 PMC10178747

[116] Shen Z, Shan X, Zhang S, Huang D, Hu H, Liang Y, et al. Alpinetin targets mitophagy pathways to mitigate Parkinson’s disease progression. CNS Neurosci Ther. 2025;31(12):e70676. 41331739 10.1002/cns.70676 PMC12672210

[117] Lee YT, Huang SQ, Lin CH, Pao LH, Chiu CH. Quantification of gut microbiota dysbiosis-related organic acids in human urine using LC-MS/MS. Molecules. 2022;27(17):5363. 36080134 10.3390/molecules27175363 PMC9457824

[118] Sobolev PD, Burnakova NA, Beloborodova NV, Revelsky AI, Pautova AK. Analysis of 4-hydroxyphenyllactic acid and other diagnostically important metabolites of alpha-amino acids in human blood serum using a validated and sensitive ultra-high-pressure liquid chromatography-tandem mass spectrometry method. Metabolites. 2023;13(11):1128. 37999224 10.3390/metabo13111128 PMC10673366

[119] Herebian D, Seibt A, Smits SHJ, Rodenburg RJ, Mayatepek E, Distelmaier F. 4-Hydroxybenzoic acid restores CoQ(10) biosynthesis in human COQ2 deficiency. Ann Clin Transl Neurol. 2017;4(12):902–8. 29296619 10.1002/acn3.486 PMC5740244

[120] Hurtado-Barroso S, Quifer-Rada P, Marhuenda-Munoz M, Rinaldi de Alvarenga JF, Tresserra-Rimbau A, Lamuela-Raventos RM. Increase of 4-hydroxybenzoic, a bioactive phenolic compound, after an organic intervention diet. Antioxid (Basel). 2019;8(9):340. 10.3390/antiox8090340 PMC6769758 31450569

[121] Jung Y, Park J, Kim HL, Sim JE, Youn DH, Kang J, et al. Vanillic acid attenuates obesity via activation of the AMPK pathway and thermogenic factors in vivo and in vitro. FASEB J. 2018;32(3):1388–402. 29141998 10.1096/fj.201700231RR

[122] Park WY, Park J, Ahn KS, Kwak HJ, Um JY. Ellagic acid induces beige remodeling of white adipose tissue by controlling mitochondrial dynamics and SIRT3. FASEB J. 2021;35(6):e21548. 33956354 10.1096/fj.202002491R

[123] Uhlen M, Fagerberg L, Hallstrom BM, Lindskog C, Oksvold P, Mardinoglu A, et al. Tissue-based map of the human proteome. Science. 2015;347(6220):1260419. 25613900 10.1126/science.1260419

[124] Human Protein Atlas. Protein Atlas v18.0: Protein classes [Internet]. Available from: https://v18.proteinatlas.org/humanproteome/proteinclasses

[125] Wright FA, Lemon WJ, Zhao WD, Sears R, Zhuo D, Wang JP, Yang HY, Baer T, Stredney D, Spitzner J, et al. A draft annotation and overview of the human genome. Genome Biol. 2001;2(7):RESEARCH0025. 11516338 10.1186/gb-2001-2-7-research0025 PMC55322

[126] UniProt C. UniProt: the universal protein knowledgebase in 2025. Nucleic Acids Res. 2025;53(D1):D609–17. 39552041 10.1093/nar/gkae1010 PMC11701636

